# Brain Research to Ameliorate Impaired Neurodevelopment - Home-based Intervention Trial (BRAIN-HIT)

**DOI:** 10.1186/1471-2431-10-27

**Published:** 2010-04-30

**Authors:** Jan L Wallander, Elizabeth McClure, Fred Biasini, Shivaprasad S Goudar, Omrana Pasha, Elwyn Chomba, Darlene Shearer, Linda Wright, Vanessa Thorsten, Hrishikesh Chakraborty, Sangappa M Dhaded, Niranjana S Mahantshetti, Roopa M Bellad, Zahid Abbasi, Waldemar Carlo

**Affiliations:** 1 Psychological Sciences and Health Sciences Research Institute, University of California, Merced, 5200 Lake Road, Merced, CA 95343, USA; 2 Department of Statistics and Epidemiology, RTI International, 3040 Cornwallis Road, Durham, NC 27709, USA; 3 Department of Psychology, University of Alabama at Birmingham, 1530 3rd Avenue South, Birmingham, AL 35294, USA; 4 Departments of Physiology and Medical Education, J N Medical College, Belgaum, 590 010 Karnataka, India; 5 Departments of Community Health Sciences and Family Medicine, Aga Kahn University, Stadium Road, Karachi 74800, Pakistan; 6 University Teaching Hospital, Private Bag RW1X, Lusaka, Zambia; 7 Department of Public Health, Western Kentucky University, 1906 College Heights Blvd, AC 129A, Bowling Green, KY 42101, USA; 8 Center for Research for Mothers and Children, Eunice Kennedy Shriver National Institute of Child Health and Human Development, 6100 Executive Blvd., Room 4B05J, Rockville, MD 20852, USA; 9 Department of Pediatrics, J. N. Medical College, Belgaum, 590 010 Karnataka, India; 10 Division of Neonatology, Department of Pediatrics, University of Alabama at Birmingham, 619 South 20th Street, Birmingham, AL 35233-7335, USA

## Abstract

**Background:**

This randomized controlled trial aims to evaluate the effects of an early developmental intervention program on the development of young children in low- and low-middle-income countries who are at risk for neurodevelopmental disability because of birth asphyxia. A group of children without perinatal complications are evaluated in the same protocol to compare the effects of early developmental intervention in healthy infants in the same communities. Birth asphyxia is the leading specific cause of neonatal mortality in low- and low-middle-income countries and is also the main cause of neonatal and long-term morbidity including mental retardation, cerebral palsy, and other neurodevelopmental disorders. Mortality and morbidity from birth asphyxia disproportionately affect more infants in low- and low-middle-income countries, particularly those from the lowest socioeconomic groups. There is evidence that relatively inexpensive programs of early developmental intervention, delivered during home visit by parent trainers, are capable of improving neurodevelopment in infants following brain insult due to birth asphyxia.

**Methods/Design:**

This trial is a block-randomized controlled trial that has enrolled 174 children with birth asphyxia and 257 without perinatal complications, comparing early developmental intervention plus health and safety counseling to the control intervention receiving health and safety counseling only, in sites in India, Pakistan, and Zambia. The interventions are delivered in home visits every two weeks by parent trainers from 2 weeks after birth until age 36 months. The primary outcome of the trial is cognitive development, and secondary outcomes include social-emotional and motor development. Child, parent, and family characteristics and number of home visits completed are evaluated as moderating factors.

**Discussion:**

The trial is supervised by a trial steering committee, and an independent data monitoring committee monitors the trial. Findings from this trial have the potential to inform about strategies for reducing neurodevelopmental disabilities in at-risk young children in low and middle income countries.

**Trial Registration:**

Clinicaltrials.gov NCT00639184

## Background

### Research justification

Birth asphyxia, or failure to initiate spontaneous respiration, is the leading specific cause of neonatal mortality in low- and low-middle income countries (L/LMIC) and accounts for about one million the four million neonatal deaths that occur each year worldwide [1]. Birth asphyxia is also the main cause of neonatal encephalopathy [1-3] and long-term morbidity including intellectual disability, cerebral palsy, and other neurodevelopmental disorders [2-4]. Mortality and morbidity from birth asphyxia disproportionately affect more infants in L/LMIC, particularly those from the lowest socioeconomic groups [5]. Therefore, neonatal resuscitation is being implemented through formal training programs in many L/LMIC [6,7]. Although improving resuscitation in L/LMIC may save lives, this intervention may also increase the number of survivors at risk for neurodevelopmental impairment. This has not been evaluated well in L/LMIC. However, prior empirical findings suggest that low cost, home-based, parent-provided early developmental interventions can reduce this risk and improve developmental outcomes in children [8].

### Sequelae of birth asphyxia

Birth asphyxia can result in hypoxic-ischemic brain injury that selectively affects vulnerable areas of the immature brain. The type of neurodevelopmental sequelae, as determined by the specific area of the brain damaged and by the severity, duration, and timing of the insult, can include cognitive disabilities such as mental retardation, motor disabilities, and visual and hearing impairment [e.g, [4,9]]. Even in the absence of mental retardation, children surviving birth asphyxia may display general cognitive abilities in the lower range of normal and learning disabilities. Sociocultural factors found in L/LMIC, such as poverty, illiteracy, low status of women, poor hygiene, lack of clean water and sanitation, and inadequate access to health care and social services exacerbate the risk for neurodevelopmental disability following birth asphyxia [10].

### Early developmental intervention

Programs of early developmental intervention (EDI) are capable of preventing or limiting the declines in cognitive development that may occur in the first years of life following brain insult. EDI has been defined as a broad array of activities designed to enhance a young child's development [11]. EDI interventions are intended to affect children directly via structured experiences and indirectly through influencing the care giving environment. These services can be provided in a variety of contexts, including home, center, hospital, and/or clinic, with the intensity of the interventions being based on the child's and family's needs and resources, the cultural milieu, and access to trained professionals. The primary goals for EDI include improving the child's developmental trajectories and assisting the family in addressing the needs of a child with, or at risk for, developmental delays.

The positive effects of EDI on early child development have been demonstrated in numerous controlled trials in the US and other high-income countries [e.g., [12-17]], which have been confirmed through meta-analyses [18,19] as well as expert reviews [e.g., [16,20,21]]. The average effect size across studies evaluating EDI has ranged from 0.50 - 0.75 [21,22].

### Early developmental intervention in L/LMIC

The effects of EDI on early child development in infants with birth asphyxia in L/LMIC have not been evaluated sufficiently to determine if these interventions should become standard practice. We are aware of only one small single-center randomized controlled trial targeting the risk group of infants with birth asphyxia. Conducted in China [23], the effects of EDI were evaluated in 64 infants with birth asphyxia, who were randomized to EDI (n = 34) or conventional care (n = 30). A group of term infants (n = 38) without birth asphyxia was also provided conventional care for comparison. A relatively low-intensity home-based, parent-provided intervention included home visits to teach the parents how to provide stimulating activities with their infants. Following two visits in the first month, subsequent home-visit were made monthly during the first year and then every two months in the second year, when parents received continued instruction to apply age-appropriate stimulation activities with their infants.

Neurodevelopmental assessment performed by a masked trained evaluator using a modified Bayley Scales of Infant Development (BSID) [24] at 18-24 months, documented mental development index (MDI) for asphyxiated infants of 105 ± 15 in the EDI group vs. 91 ± 11 in the conventional care group (*p *< 0.001). Normal term infants who received conventional care and obtained a MDI of 100 ± 13. The BSID psychomotor development index (PDI) did not differ significantly between the groups. This trial suggests that a program of EDI, at a level of intensity lower than evaluated in high-income countries, may effectively promote cognitive development in infants with birth asphyxia. The effect size was about 1.0 standard deviation (SD). Nonetheless, it is unknown if the cognitive benefits are reproducible and generalizable because of the limited scope of the trial and the small sample size.

Several trials of EDI with other risk groups of infants and young children conducted in different L/LMIC, uniformly document its positive effects on child development. This has included groups at risk for neurodevelopmental disability due to *prematurity or low birth weight *in China [25] and Jamaica [26,27]; *growth retardation or stunting *in Jamaica [28-30]; *malnourishment *in Colombia [31,32], Jamaica [33-36], India [37], and Bangladesh [38,39]; as well as *socioeconomic deprivation *in Jamaica [40], Brazil [41] and Vietnam [42]. Follow-up evaluations as long as 18 years have shown significant positive effects for those who received EDI during the first two years of life on measures of intelligence, reading comprehension, mental health, and self-esteem [43,44].

While several studies have suggested positive effects on the development of children at risk for neurodevelopmental disabilities in a number of L/LMIC, most enrolled relatively small samples. Thus, a larger, multi-site study is needed to evaluate EDI in L/LMIC.

### Background information on the trial

The Brain Research to Ameliorate Impaired Neurodevelopment - Home-based Intervention Trial (BRAIN-HIT) was conducted in communities in India, Pakistan, and Zambia that participated the Global Network for Women's and Children's Health Research and are sites of the FIRST BREATH (FB) Trial. The FB Trial was the first randomized trial to test the effectiveness of standard educational programs for resuscitation interventions (initial steps of resuscitation and bag and mask ventilation) to reduce mortality from birth asphyxia (ClinicalTrials.gov). The FB Trial was developed to answer the question of whether increased resuscitation efforts would be associated with an increase of survivors at-risk for neurodevelopmental disability in societies least able to provide for their care. Therefore, BRAIN-HIT is a follow-up trial of a sub-set of the FB Trial infants who were resuscitated by bag and mask ventilation. BRAIN-HIT is designed to evaluate whether EDI can improve neurodevelopment in infants at-risk for poor neurodevelopment outcome due to birth asphyxia.

### Trial sites

BRAIN-HIT is implemented at sites in India (Jawaharlal Nehru Medical College, Belgaum), Pakistan (The Aga Khan University, Karachi), and Zambia (University Teaching Hospital/University of Zambia School of Medicine, Lusaka). In addition, RTI International serves as the data coordinating center for the trial. Sites in India, Pakistan, and Zambia were selected for the BRAIN-HIT because these countries have well organized primary care health systems yet a high proportion of home deliveries attended by traditional birth attendants and a majority of home deliveries. Birth asphyxia is a major cause of neonatal mortality and neurodevelopmental disability in all three countries. The study sites in India, Pakistan and Zambia are relatively rural and have a high perinatal mortality rate. The India site was located in Karnataka State; in Pakistan, the site was in Thatta, about 100 kilometers from Karachi; and in Zambia, the site was located in Chongwe and Kafue Districts, about 100 kilometers from Lusaka.

### Formative and feasibility research

Prior to embarking on BRAIN-HIT, investigators tested the feasibility of the methods over a two-year period in Zambia. Funded with a planning grant from the NIH Fogarty International Center, pilot work included perinatal data collection, identification of infants with birth asphyxia (Ellis neurological exam and Apgar scoring), recruitment of at risk infants, administration of the EDI program, and neurodevelopmental testing. Indicated modifications in the methods were made before implementation of the current RCT.

### Aim, objectives, and hypotheses

The *overall aim *of BRAIN-HIT is to evaluate the effects of an EDI program on the development of children in L/LMIC who are at-risk for neurodevelopmental disability because of birth asphyxia. A group of children without perinatal complications are evaluated in the same protocol to compare the effects of the EDI in healthy infants in the same communities. If the study shows positive results, the long-term aim is to empower local health systems with training and logistic support to sustain an EDI program that can reduce morbidity for children in L/LMIC with high rates of birth asphyxia as well as stimulate further research and implementation of comparable programs worldwide.

The *specific objectives *are to: (1) Evaluate the effects of a home-based EDI plus health and safety counseling (HSC) compared to HSC alone on the cognitive, social-emotional, and motor development at 12, 24 and 36 months in children with birth asphyxia; (2) Determine whether children with birth asphyxia who receive EDI demonstrate cognitive, social-emotional and motor development at 12, 24 and 36 months of age that is indistinguishable from that of children without perinatal complications from the same communities; and (3) Examine whether the effects of EDI on children with birth asphyxia are moderated by child, parent, and family characteristics.

The *primary hypothesis *is that home-based EDI and HSC will improve cognitive development (as indicated by a difference in BSID MDI scores of 10 points [0.67 SD]) at 36 months of age compared to HSC only, in children with mild to moderate birth asphyxia and normal post-natal neurological exam. This hypothesis is also tested in groups of children without perinatal complications from the same communities. The following *secondary hypotheses *are also tested in children with and without birth asphyxia: (1) Improvements will be observed in children who receive EDI in social-emotional and motor development at 36 months of age compared to those who do not; (2) Improvements will be observed in children who receive EDI in all developmental domains at 12 and 24 months of age; compared to those who do not (3) The improvements in cognitive, social-emotional, and motor development related to EDI will be larger in the children with birth asphyxia compared to those without perinatal complications; (4) The effects of EDI in children with and without birth asphyxia will be moderated by child, parent, and family characteristics; and (5) Children who receive a higher EDI dose (i.e., are exposed to more EDI home visits by a parent trainer) will experience greater developmental improvements than those who receive a lower dose.

## Methods/Design

### Design

The study sample for this RCT was drawn from two populations. The birth asphyxia group had mild-to-moderate birth asphyxia, was resuscitated at birth, and had a neurological examination consistent with normal or Stage I-II on the Ellis scale in the first week of life. The group without birth asphyxia constituted a comparison group of children who were not resuscitated and had experienced no perinatal complications. Within each group, children and their mothers were randomized to either the intervention arm (EDI + HSC) or the control arm (HSC only).

To ensure that any effect of the intervention was related to the curriculum used in the home visits of the EDI, rather than simply to having a number of visits from a provider, parents in the control condition receive visits on the same schedule (every two weeks) throughout the three years of the trial as those in the intervention arm. However, only HSC is provided to participants in the control condition. This intervention trial cannot be fully masked, as the parents and the parent trainers are aware of which condition they are in, but to reduce bias, the evaluators administering the 12, 24 and 36 month assessments are blinded to the treatment assignments.

### Randomization

The data center generated randomization lists for infants with and without birth asphyxia, using a block randomization procedure (randomization occurred within blocks of 4 or 8). Each site was provided with sealed envelopes with the randomization assignment. For each randomized infant with birth asphyxia, the sites then randomized the next one to two infants without birth asphyxia so that an overall ratio of approximately 1-to-2 was achieved.

### Interventions

#### Experimental: Early Developmental Intervention

A home-based, parent-implemented EDI model was chosen because the home is the foremost natural environment for learning to occur for a child aged birth to three, and the parent (or the equivalent) is one of the limited numbers of caring persons with whom the infant or toddler is attached [11,21]. This approach supports the parent in the role as the first teacher of the child and provides opportunities for strengthening the parent-child bond. This model is also especially well suited for low-resource settings because it requires relatively little infrastructure and resources to implement compared to alternative model, such as center-based interventions. The EDI will be provided over the first 36 months of children's lives. While the intervention in the vast majority of cases will target the mother, fathers as well as extended family members are welcomed additional participants.

#### Curriculum

The *Partners for Learning *[45] curriculum and supplemental materials provide the structure and substance of the EDI. This curriculum served as the core intervention in two of the first large-scale RCT evaluations of EDI in the U.S. [17,46], which demonstrated significant cognitive, language, and social gains and long-lasting improvement in educational achievement and social outcomes for children at risk for developmental problems who participated in this program. In addition, research has shown that the *Partners for Learning *curriculum can be implemented effectively in home visitation programs for children at high risk due to low birth weight [13,47].

*Partners for Learning *has several components that are transmitted via a trainer who visits parents in the children's homes. During each visit, the trainer presents playful interactive learning activities, which are depicted on cards. Each activity targets a developmentally appropriate competence Cycles of use allow the parent to implement several activities for a while, but then move on to new activities as the child masters each competence. This progression is guided by the trainer, who selects activities to match and enhance the child's developmental competences. *Partners for Learning *covers a full spectrum of 23 developmental skill areas, organized into the four areas: (1) cognitive and fine motor, (2) social and self-help, (3) gross motor, and (4) language skills. The trainer encourages the parent to apply the targeted activities until the next home visit by integrating them into daily life with the child. The activities can thus enrich care routines such as diapering, feeding, dressing and special one-to-one times. By applying these activities and observing how the child changes and acquires these competences, general principles gradually emerge that enable the parent to gain a deeper understanding of early child development. With this understanding, the parent can appreciate her own important contribution to the child's development, thereby gain enhanced efficacy in the parenting role and become empowered as an important agent in the child's life.

#### Staff, training, and implementation

Each site selected EDI trainers and supervisors with appropriate backgrounds to its context, such as education, nursing, public health, and physiotherapy. The trainers conducting the EDI home visits and the supervisors initially participated in a five-day workshop conducted at each research site by experienced EDI professionals from the U.S.. The training covered expectations, rationale, early child development, home visiting procedures, the *Partners for Learning *curriculum and materials, assessment and monitoring of progress, and partnering with parents. Also the Portage Model of home visiting [48] was stressed as a way for the EDI trainers to structure their approach to working with mothers (and other family members) during each visit. The training included practice and feedback. A second workshop was conducted at each site when participating children began to reach age 18 months. While some of the same material was reviewed as at the first workshop, this workshop focused on adapting EDI procedures for the children as they develop and observing trainers in actual home visits, to provide constructive feedback.

Each parent-child pair has an assigned trainer conducting the home visits, which are scheduled to occur every two weeks. A form is used to record each visit and which activities were addressed. One to two activities are taught to the parent at each visit. The trainer demonstrates the activity with the child and explains its value. The parent then practices the activity with the child and is given encouraging feedback to fine-tune her interaction with the child. The parent is given the card demonstrating each activity that has been addressed in the visit and is instructed to apply the activities as frequently as possible in her everyday interactions with the child until the next visit.

#### Control: Health and Safety Counseling

A modified version of the World Health Organization health education curriculum http://www.who.int constituted the Health and Safety Counseling (HSC) curriculum used in both arms of the trial. A range of health and safety issues are addressed over time, for example breastfeeding, nutrition, hygiene, safety in the home and community, management of child diarrhea, and well-child check-ups and vaccinations. This material is discussed with the parent during home visits on the same schedule as the EDI. While participants in the experimental condition also receive HSC in addition to EDI, those in the control condition receive only HSC.

### Duration

Both the experimental and control conditions are implemented over the child's first 36 months of life, in the form of home visits every two weeks. Each intervention visit is designed to take 30 to 45 minutes. The developmental and health assessments to address the hypotheses are conducted at child ages 12, 24, and 36 months. These assessments generally take two to three hours.

### Enrollment

The primary population is children born in the delivery services of the health care centers/hospitals in Lusaka, Zambia or in rural communities in India, Pakistan, and Zambia participating in the FB Trial. Specific eligibility criteria included: a child with (1) birth asphyxia defined as requiring bag-and-mask resuscitation; (2) birth weight ≥ 1,500 g; (3) neurological examination consistent with mild or moderate (Stage I or II) status on the Ellis scale (Ellis et al. 2000); and (4) a parent who is willing to participate in an intervention program for 36 months. The few children who survive severe birth asphyxia and have severely impaired neurological examinations (Ellis Stage III) are excluded because they have a very high infant mortality rate and are unlikely to benefit from EDI [49]. A healthy comparison group of children without birth asphyxia or other perinatal complications (Apgar > 6 at 5 minutes) and with a birth weight ≥ 1500 g and Ellis Stage I or II rating during the first week are recruited from the same delivery centers and communities over the same time span. All enrolled participants complete approved informed consent procedures. Parents are compensated for travel and the time taken and wages lost to complete the 12, 24, and 36 month evaluations.

### Power analysis

The primary hypothesis is that a home-based EDI plus HSC compared to HSC alone will improve the BSID MDI at 36 months by 10 points in children with birth asphyxia. At least 40 participants per condition who complete the 36 month assessment in each randomization group (birth asphyxia and healthy comparison group) will be needed to be able to detect the hypothesized difference of 10 MDI points (= 0.67 SD) between the experimental and control condition with 5% level of significance and 90% power [50,51]. Assuming 10% attrition per year and possible missing values, at least 60 children per condition and group, or at least 120 children with birth asphyxia and 120 healthy comparison children for a total of 240, need to be enrolled and randomized to either EDI plus HSC or HSC alone to achieve the desired power.

Consequently, at least 80 children (40 in each group) were to be enrolled in each country. However, due to anticipated higher attrition in the healthy comparison group, the sites were to enroll additional children from this group. In total, 431 children across the two groups have been enrolled and randomized during the defined enrollment period, including 174 in the birth asphyxia and 257 in the healthy comparison group.

### Measures for testing hypotheses

The following measures are collected at child ages 12, 24, and 36 months. In addition, the Parent Attitudes and Knowledge Inventory and a family demographics and resources measures were administered at enrollment prior to neonatal age 2 weeks. The parent-report instruments were translated into the appropriate language and are always administered in a standardized structured interview to address significant literacy variation among the parent participants. All instruments are administered by blinded evaluators, who were trained in annual workshops and monitored for quality throughout.

#### Bayley Scales of Infant Development

The Bayley Scales of Infant Development II (BSID) [24] measures both the primary outcome variable, cognitive development in the form of the MDI, and a secondary outcome variable, physical development in the form of the PDI. The BSID is a well-validated measure of development in the first 3.5 years and is commonly used to evaluate EDI programs in high-income countries. However it is not routinely used in L/LMIC. Therefore, extensive testing, some cultural adaptation, thorough training on three occasions throughout the trial, and ongoing monitoring of the evaluators is central to the study. The evaluators administer the BSID directly to each child in the appropriate language using standard material prescribed in the manual.

#### Ages & Stages Questionnaire

The Ages and Stages Questionnaire, 2^nd ^ed. (ASQ) [52] is a 30-item instrument addressing parent-reported child development in the domains of communication, gross motor, fine motor, problem solving, and personal-social development using age-specific forms. It is used in BRAIN-HIT to provide a secondary measure of general child development observed in the home environment. To reduce the impact of variation in literacy, ASQ is administered in an interview format with the parent, who is instructed to report on the child's behavior observed in the home or community. As such, the ASQ complements the information on cognitive development obtained through direct testing with the BSID. The ASQ has been widely used for the assessment of early development, including in numerous L/LMICs [e.g., [53,54]].

#### Ages & Stages Questionnaire: Social-Emotional

The Ages and Stages Questionnaire: Social and Emotional (ASQ:SE) [55] uses between 22-30 items (depending on age) to assess parent-reported child social and emotional functioning in the areas of self-regulation, compliance, communication, adaptive functioning, autonomy, affect, and interaction with people. It is used to measure social-emotional development, a secondary outcome in this research. Except for the specific content, the ASQ:SE is formatted and used in the same manner as the ASQ.

#### Parent Attitudes and Knowledge Inventory

Parent attitudes regarding developmental expectations, contributions towards development, and efficacy in the parenting role are measured with 16 items adapted from other longer and more detailed parent attitude instruments [56-58]. Parent knowledge about early child development is measured with another 16 items adapted from the Knowledge of Infant Development Inventory [59], a 75-item instrument designed to assess parents' factual knowledge of parental practices, child developmental processes, and infant norms of behavior.

#### Child health

The primary health measures are weight, height, and head circumference; screened vision and hearing impairment and severe malnutrition; and immunization status. Additional indicators of health to be determined will be collected at ages 24 and 36 months.

#### Family demographics and resources

Family demographics and economic resources are measured with a modified World Bank tool addressing maternal age, race, and education; family size and assets; and household utilities. In addition, maternal psychosocial resources are measured with 12 items developed for this project to address frequency of her interactions with family members and other women as well as time to manage her life and demands.

#### Intervention dose

The parent trainers maintain detailed records on each home visit made, including date, duration, activities addressed, and reason for missing (if applicable), from which various dose-relevant measures will be calculated. The primary indicator of intervention dose will be number of home visits made per 12-month period.

### Data management

Data from forms completed by evaluators or parent trainers are entered locally into a digital database with range and consistency checks. These data are periodically transmitted electronically to the data coordinating center (DCC; RTI International, Research Triangle Park, NC, USA) where additional data edits, including inter- and intra-form consistency checks, are performed. Descriptive statistics are generated and reported to investigators on a monthly basis to monitor enrollment and retention, completion of home visits, and rescheduled or missed visits, and completion of 12, 24 and 36 month evaluations. Additionally, the study sites receive regular edit reports and reconcile any missing, incomplete, or inconsistent data.

### Data analysis

The primary outcome for the study will be analyzed using an intent-to-treat strategy, where data for all participants who meet the inclusion/exclusion criteria will be included in the analysis, regardless of how many home visits are completed. Secondary analyses will be conducted to evaluate the impact of the adherence to the study protocol (e.g., number of visits) on the relationship of the intervention to study outcomes. Because of high post-neonatal childhood mortality (5%), particularly in the group with asphyxia, some attrition is expected.

Comparisons of MDI between the two trial conditions at the end of the study will employ student's t-test, which are repeated for the birth asphyxia and health comparison groups. Descriptive statistics will be reported as frequencies and percentages for categorical variables and means, standard deviations, and minimum and maximum values for continuous variables. Additionally, generalizing estimating equations models and hierarchical linear modeling will be used to perform the analysis on the primary and secondary outcome variables. Other secondary data analyses involving continuous outcomes will be performed using multivariate analysis. Simple ordinary regression models for continuous models will be used to analyze within treatment group differences in outcomes.

To determine cost-effectiveness, the incremental cost of sustaining a home-based early intervention program will be compared to the "willingness to pay" for a disability-adjusted life year (DALY). Maintenance of a home-based intervention program has a relatively low incremental cost from a programmatic standpoint. The incremental cost per year once the program is established will be the salary of a full time trainer, training equipment, transportation, and supplies. For example, experimental research in Lusaka, Zambia has shown that if the rate of MDI <70 due to birth asphyxia is reduced from 7 to 3.5%, the incremental cost will be around $8.50 per DALY ($22,000 ÷ [3.5/100 absolute risk reduction × 2000 survivors of asphyxia enrolled per year × 37 years life expectancy]). This cost compares favorably with that of other medical procedures [60].

## Discussion

### Administrative structures - Trial Steering Committee

The Trial Steering Committee (TSC) consists of all principal investigators and the supervising trial manager. Statisticians and field investigators join the TSC deliberations as needed. The TSC met initially to finalize the protocol and organization of the trial. The TSC then meets regularly to discuss issues such as recruitment progress, trial implementation data, and indications of protocol deviations. The TSC evaluates this information and take action accordingly. It also plans the training of parent trainers and evaluators.

### Research ethics

The study was reviewed and approved by the Institutional Review Boards (IRBs) at all participating sites in India, Pakistan, and Zambia, as well as at the central U.S. sites at the University of Alabama at Birmingham and the Research Triangle Institute (RTI). The study has been reviewed and approved also by the NICHD Global Network for Women's and Children's Health Research data monitoring committee (DMC). The primary publication for the trial will follow CONSORT guidelines for randomized controlled trials. Criteria for authorship of all papers, presentations, and reports resulting from the study will conform to ICMJE standards.

### Data and process monitoring

Adverse events for the trial, including the death of a child or mother or hospitalization of the child, are reported to the DMC. Reports and recommendations from the DMC are distributed to the IRBs and the project officer representing the funding agency (NICHD). Process monitoring and management is directed by the international site investigators and includes: (1) assuring high compliance rates with the intervention visits (>80%); (2) evaluating accuracy and completeness of data collected, entered, and transmitted (jointly with the DCC); (3) assuring that all personnel are fulfilling their obligations; (4) addressing ad hoc problems and maintaining communication; and (5) maintaining proper IRB approval. The DCC also assures inter-site consistency. Data on knowledge are collected before and immediately after each staff training session. Parent trainer competency is observed and evaluated with a checklist by a supervisor at one home visit per six months.

### Implications

Birth asphyxia (defined as inability of a neonate to establish spontaneous breathing after birth) is a major cause of long-term neurodevelopmental morbidity, such as intellectual disability and cerebral palsy, and affects a disproportionate number of children in L/LMIC. EDI has shown in numerous prior RCTs in L/LMIC to be capable of preventing or limiting the declines in cognitive development that may occur in the early years of life in children following brain insult or who are otherwise at risk for developmental problems. The findings from the current RCT therefore have the potential to identify strategies for reducing neurodevelopmental disabilities in at-risk young children in L/LMIC that require relatively low resources.

## Abbreviations

ASQ: Ages & Stages Questionnaire; ASQ: SE Ages & Stages Questionnaire: Social-Emotional; BRAIN-HIT: Brain Research to Ameliorate Impaired Neurodevelopment - Home-based Intervention Trial; BSID: Bayley Scales of Infant Development; CONSORT: Consolidated Standards of Reporting Trials; DALY: Disability-Adjusted Life Year; DCC: Data Coordination Center; DMC: Data Monitoring Committee; EDI: Early Developmental Intervention; FB FIRST BREATH: HSC Health and Safety Counseling; ICMJE: International Committee of Medical Journal Editors; IRB: Institutional Review Board; L/LMIC: Low and Low-Middle Income Countries; MDI: Mental Development Index; NICHD: National Institute of Child Health and Human Development; NIH: National Institutes of Health; PDI: Physical Development Index; RCT: Randomized Controlled trial; TSC: Trial Steering Committee.

## Competing interests

This project has been funded by the Fogarty International Center and is currently being funded by the Eunice K Eunice Kennedy Shriver National Institute on Child Health and Human Development, both of the National Institutes of Health in the U.S.. The authors of this paper have no competing interests to declare.

## Authors' contributions

WC, JW, FB, and EM conceived of and designed the study, obtained funding, and designed the intervention and evaluation procedures. SG, OP, EC, DS, and LW participated in the design of the study and the intervention and evaluation procedures. VT and HC participated in the study design, data management, and statistical analysis. SD, NM, RB, and ZA participated in the design and implementation of the evaluation. The remaining members of the BRAIN-HIT Investigators group participated in the design of the study. All authors have read and approved of the manuscript.

## Pre-publication history

The pre-publication history for this paper can be accessed here:

http://www.biomedcentral.com/1471-2431/10/27/prepub
